# Effect of Transcranial Brain Stimulation for the Treatment of Alzheimer Disease: A Review

**DOI:** 10.1155/2012/687909

**Published:** 2011-10-25

**Authors:** Raffaele Nardone, Jürgen Bergmann, Monica Christova, Francesca Caleri, Frediano Tezzon, Gunther Ladurner, Eugen Trinka, Stefan Golaszewski

**Affiliations:** ^1^Department of Neurology, Christian Doppler Clinic, Paracelsus Medical University, 5020 Salzburg, Austria; ^2^Department of Neurology, Franz Tappeiner Hospital, Via Rossini 5, 39012 Meran/o, Italy; ^3^Neuroscience Institute, Christian Doppler Clinic, 5020 Salzburg, Austria; ^4^Department of Physiology, Medical University of Graz, 8010 Graz, Austria

## Abstract

Available pharmacological treatments for Alzheimer disease (AD) have limited effectiveness, are expensive, and sometimes induce side effects. Therefore, alternative or complementary adjuvant therapeutic strategies have gained increasing attention. The development of novel noninvasive methods of brain stimulation has increased the interest in neuromodulatory techniques as potential therapeutic tool for cognitive rehabilitation in AD. In particular, repetitive transcranial magnetic stimulation (rTMS) and transcranial direct current stimulation (tDCS) are noninvasive approaches that induce prolonged functional changes in the cerebral cortex. Several studies have begun to therapeutically use rTMS or tDCS to improve cognitive performances in patients with AD. However, most of them induced short-duration beneficial effects and were not adequately powered to establish evidence for therapeutic efficacy. Therefore, TMS and tDCS approaches, seeking to enhance cognitive function, have to be considered still very preliminary. In future studies, multiple rTMS or tDCS sessions might also interact, and metaplasticity effects could affect the outcome.

## 1. Introduction

Given the limited efficacy of pharmacological treatments [1], nonpharmacological approaches in AD are of great interest. In recent years, new techniques for studying the human brain that allow for the noninvasive neurostimulation have emerged. In particular, two techniques of noninvasive brain stimulation—repetitive transcranial magnetic stimulation (rTMS) and transcranial direct current stimulation (tDCS)—are capable for modulating cortical excitability and inducing lasting effects [2, 3]; both have been shown to have potential therapeutic efficacy in cognitive neuroscience [4]. By using rTMS, depending on the location and the stimulation parameters as well as on the physiology of the underlying cortical tissue, behavioural changes may also be seen, including enhancement of or interference with cognitive performance [5, 6]. rTMS has been increasingly utilized for various neurological [7–9] and psychiatric conditions [10–12]. tDCS has also been shown to induce cognitive improvements in healthy subjects [13–15] and patients with neuropsychiatric disorders such as depression [16–18], Parkinson disease [19, 20], and stroke [21]. Both neuromodulatory techniques can induce lasting modulation of brain activity in the targeted brain region and across brain networks through transcranial induction of electric currents in the brain [4]. In the last years, these two neuromodulatory techniques have been proposed as a possible treatment to improve cognitive performances, also in subjects affected by dementia in which it may represent a useful tool for cognitive rehabilitation.

## 2. Therapeutic Interventions

### 2.1. Neuromodulatory Techniques

rTMS is a technique that delivers single TMS pulses in trains with a constant frequency and intensity for a given time. tDCS is another simple and powerful tool to modulate brain activity, which delivers low-intensity electrical currents (below the perceptual threshold, 1 to 2 mA) over the scalp using two large saline-soaked sponge electrodes. The resulting constant electrical field penetrates the skull and influences neuronal function.

rTMS can be applied as continuous trains of low-frequency (1 Hz) or bursts of higher-frequency (≥5 Hz) rTMS, while tDCS can be applied as anodal or cathodal stimulation [4]. In general, low-frequency rTMS and cathodal tDCS are thought to reduce, and high-frequency rTMS and anodal tDCS to enhance excitability in the targeted cortical region. However, it is not completely understood by which mechanisms of action rTMS and tDCS can induce lasting effects on the brain. The physiologic impact of both techniques involves synaptic plasticity, specifically long-term potentiation (LTP) and long-term depression (LTD). A link between the aftereffects induced by rTMS and the induction of synaptic plasticity has been recently identified [22]. Similarly, tDCS may modulate synaptic strength within the cortex, with evidence pointing to the involvement of intracortical neurons [23].

### 2.2. Repetitive Transcranial Magnetic Stimulation

A recent meta-analysis of publications searching for the effects of rTMS on cognitive functions [24] found convincing data supporting improvement in several cognitive functions, including executive functions, learning, and memory.

It has been demonstrated in elderly subjects that rTMS induces a transient improvement in the associative memory task and that it is associated with recruitment of right prefrontal and bilateral posterior cortical regions [25]. Three studies have been carried out to assess the effects of rTMS on naming and language performance in patients with probable AD. 

In two crossover, sham-controlled, single-session studies [26, 27] rTMS was applied to the dorsolateral prefrontal cortex (DLPFC) during the execution of naming tasks (on-line rTMs). In the first study, a significantly improved accuracy in action naming, but not in object naming, was found following high-frequency rTMS of either left or right DLPFC in each of the 15 examined patients [26]. In the second study [27], the effect of rTMS applied to the DLPFC on picture naming was assessed in 24 AD patients with different degrees of cognitive decline. The authors found that the results of the previous study were replicated only in mild AD patients (Mini-Mental-State-Examination (MMSE) ≥17/30); in contrast, in patients with moderate to severe AD (MMSE < 17/30), both action and object naming were facilitated after both left and right DLPFC rTMS. The lack of effects of rTMS on object naming in early-stage AD might be related to a “ceiling” effect; when object naming is impaired, such as in moderate to severe AD patients, rTMS to the DLPFC results in an improved performance also for this class of stimuli. The rTMS effect was bilateral both in mild and severe AD patients. The bilateral facilitation effect could be attributed to the presence of compensatory mechanisms based on the recruitment of right hemispheric resources to support the residual naming performance. 

In a later study, Cotelli et al. [28] aimed to investigate whether the application of high-frequency rTMS to the left DLPFC may lead to significant facilitation of language production and/or comprehension in patients with moderate AD. Ten patients were randomly assigned to one of two groups. The first group underwent a 4-week real rTMS stimulation protocol, while the second underwent a 2-week placebo treatment, followed by 2-weeks of real rTMS stimulation. rTMS intervention consisted of a total of 4 weeks of daily stimulation. No significant effects were observed on naming performance. However, a significant effect was observed on auditory sentence comprehension after 2 weeks of real rTMS sessions, as compared to sham. Two additional weeks of daily rTMS sessions resulted in no further improvements, but a significant benefit on auditory sentence comprehension was still detected 8 weeks after the end of the rTMS intervention. An important finding was the absence of any effects on memory and executive functions. Therefore, these results were thought to be specific to the language network, and not due to a general, nonspecific effect on cognitive processing. 

None of these three studies reports any side effects of the rTMS applications, but it is not clear what safety evaluations (if any) were completed. 

In another study, Ahmed et al. [29] aimed to compare the long-term efficacy of high- versus low-frequency rTMS, applied bilaterally over the DLPFC, on cortical excitability and cognitive function of AD patients. The high-frequency rTMS group improved significantly more than the low-frequency and sham groups in all assessed rating scales (MMSE, Instrumental Daily Living Activity Scale and the Geriatric Depression Scale) at all time points after treatment. The improvement was maintained for 3 months. The authors thus concluded that high-frequency rTMS may be a useful addition to therapy for the treatment of patients with mild to moderate degree of AD.

Since cognitive training (COG) may improve cognitive functions in AD, in a recent study Bentwich et al. [30] aimed to obtain a synergistic effect of rTMS interlaced with COG (rTMS-COG) in patients with AD. Eight patients with mild to moderate probable AD were subjected to daily rTMS-COG sessions (5/week) for 6 weeks, followed by maintenance sessions (2/week) for additional 6 months. The following six regions, located individually by MRI, were stimulated: Broca and Wernicke (language functions), right and left DLPFC (judgment, executive functions, and long-tem memory), and right and left parietal somatosensory association cortex (spatial and topographical orientation and praxias). COG tasks were developed to fit these regions. Primary outcome measures were average improvement of Alzheimer Disease Assessment Scale-Cognitive (ADAS-cog) and Clinical Global Impression of Change (CGIC); secondary objectives were average improvement of MMSE, the ADAS-Activities of Daily Living (ADAS-ADL), Hamilton Depression Scale (HAMILTON), and Neuropsychiatric Inventory (NPI). ADAS-cog improved by approximately 4 points after both 6 weeks and 4.5 months of treatment, and CGIC by 1.0 and 1.6 points, respectively. MMSE, ADAS-ADL, and HAMILTON improved, but without statistical significance, while NPI did not change. These findings provide direct evidence that rTMS is helpful in restoring brain functions and could reflect rTMS potential to recruit compensatory networks that underlie the memory-encoding and the other cognitive functions [31]. Therefore, rTMS-COG seems a promising and safe modality for AD treatment.

### 2.3. Transcranial Direct Current Stimulation

Two crossover designed studies using tDCS were performed to enhance recognition memory in patients with AD [32, 33]. Because temporoparietal areas are thought to be hypoactive in AD [34], Ferrucci et al. [32] tested in a pilot study whether anodal tDCS applied over temporoparietal cortex can increase cortical function, thus improving recognition memory in AD patients. They were delivered in 10 patients with mild AD anodal tDCS, cathodal tDCS, and sham tDCS over bilateral temporoparietal areas in three separate sessions (15 min at 1.5 mA, at least 1 week apart). Anodal tDCS significantly improved recognition memory, cathodal tDCS significantly decreased accuracy in the word recognition task, sham tDCS did not change it. Moreover, no effects were observed in a visual attention task, suggesting that the effects of tDCS were likely specific for recognition memory. Notably, no safety considerations were reported. Boggio and colleagues exposed patients with mild to moderately severe AD to a session of anodal tDCS to the left DLPFC, anodal tDCS to the left temporal cortex (cathode electrode was placed over the right supraorbital area for these 2 sessions), or a session of sham stimulation [33]. Since declarative memory is the most affected cognitive domain in AD patients, the aim of this study was to investigate the impact of anodal tDCS on recognition memory, working memory, and selective attention in AD. Sessions were 48 h apart, and patients were tested during each of the stimulation sessions, starting 10 min after stimulation onset and lasting until the end (30 min at 2 mA). Stimulation over both prefrontal and temporal areas resulted in a significant improvement of visual recognition memory, which was not attributable to a nonspecific attentional process, as assessed by the Stroop task. On contrary, no effects were obtained on working memory.

This important study has several limitations. As the authors themselves recognized, since a bipolar montage was used, it cannot be excluded that the observed effects may be the result of the stimulation from the reference electrode. Moreover, the study did not measure whether the effects of the study were long lasting, and the authors did not perform any other behavioural assessment to measure whether the effects observed in the study are clinically relevant. It should be also considered that working memory was measured by a digit span task, more indicative of attentional than working memory function.

Interestingly, Scelzo and colleagues [35] found an increased short latency afferent inhibition (SAI) after anodal tDCS in 12 healthy subjects. SAI is a TMS protocol that may give direct information about the function of some cholinergic pathways in the human motor cortex [36]. The enhancement of cortical cholinergic circuits may thus represent an important mechanism explaining anodal tDCS action in AD and several other pathologic conditions.

## 3. Discussion and Future Perspectives

The possible mechanisms that can account for the effects of rTMS and tDCS on cognitive performance conceivably reflect the potential of these methods to improve the subject's ability to relearn or to acquire new strategies for carrying out behavioural tasks. 

The use of rTMS involves the discharge of a transient electromagnetic field through the skull. Electric currents are induced in the brain by means of rapidly changing magnetic fields; in turn, these determine transsynaptic depolarizations of pools of neurons located in the superficial cortical layers. The capability of the rTMS to interact with the intrinsic abilities of the brain to restore or compensate for damaged function is a promise for possible applications in the field of cognitive rehabilitation. Since there is no clear knowledge about spatial resolution of rTMS, it is difficult to interpret the observed functional effects in terms of exact anatomical effects. The neurophysiological mechanisms responsible for rTMS-induced facilitation remain unknown, even if they are most likely related to the activation of impeded pathways or inhibition of maladaptive responses. On the other hand, rhythmic transcranial stimulation can exert positive effects on cognitive performance [37]. The modification of cortical activity through the use of rhythmic stimulation may readjust pathological patterns of brain activity, thus providing an opportunity to induce new, healthier activity patterns within the affected functional networks [38].

While rTMS elicits neuronal firing, tDCS modulates the spontaneous neuronal activity [39]. Though rTMS and tDCS both yield similar effects, tDCS has several practical advantages over rTMS. Therefore, it is simpler, safer [40], and less expensive and would be suitable for use in large series of patients, possibly even at home. In fact, the above illustrated findings prompt further studies using repeated tDCS, in conjunction with other therapeutic interventions for treating patients with AD. The prolonged aftereffects of tDCS are probably due to synaptic [41] and nonsynaptic mechanisms [42].

The effects of tDCS might be related to a facilitation of the corresponding brain area induced by the anodal electrode; this neuronal network may become, consequently, more reactive during the encoding phase of the task. The DCS is thus responsible for priming the area to receive additional behavioural intervention.

These preliminary studies highlight the therapeutic potential of the induction of long-term neuromodulatory effects using brain stimulation. They hold considerable promise, not only for advancing our understanding of brain plasticity mechanisms, but also for designing new rehabilitation strategies in patients with neurodegenerative disease. 

Therefore, rTMS and tDCS might become useful in the rehabilitation of AD patients. However, although promising, results of noninvasive stimulation to enhance cognitive function in AD to date have to be considered extremely preliminary. Most applications have been of short duration; the effects seem to be short lived and were not replicated after longer-duration interventions. Conversely, some of the effects obtained after longer-lasting interventions were not detected after single stimulation session [26–28]. Moreover, both techniques appear safe in patients with AD, even if long-term risks have been insufficiently considered.

On the other hand, the specific intervention that helps in short-term studies may not help in long-term studies. For all future studies a careful experimental design is needed and patient selection aspects, stimulation parameters, and clinical, cognitive, and behavioural assessment tools should be considered. Of great importance is a careful choice of outcome measures, also to enable comparison across studies. It would also be valuable examining therapeutic efficacy of the neuromodulatory techniques in AD to employ the neuropsychological battery of the Uniform Data Set (UDS) or outcome scales commonly used in trials of pharmacological agents for AD, such as the Cognitive subscale of the Alzheimer's Disease Assessment Scale (ADAS-Cog) [43]. Anyway, it is possible that the effects of the plasticity-based interventions with high-frequency or anodal tDCS in the brain of AD patients may differ to those in normal subjects, and studies to physiologically characterize this would be important to guide future therapeutic trials. 

It should be considered that most previous studies have failed to be evidence based, and the assumption that cortical plasticity enhancement is needed for the betterment of the cognitive status of AD patients remains conjectural [43]. 

While TMS studies showed in AD cortical hyperexcitability, the therapeutic attempts are based on techniques aimed at increasing cortical excitability (anodal tDCS, high-frequency rTMS). This hyperexcitability may be the consequence of other underlying pathophysiologic mechanisms, such as decreased synaptic efficiency or hypoplasticity. Therefore, the cortical physiology seems to require more solid investigation and should be appropriately tested before and after therapeutic interventions. On the other hand, the assumption that high-frequency rTMS will enhance cortical excitability in AD patients may be wrong. Indeed, rTMS effects dependent on the state of activity of the brain at the time of stimulation also remain debatable [44]. In addition, the assumption that in AD the mechanisms of plasticity might be abnormally reduced in the brain areas targeted in the previous studies, the DLPFC or the temporoparietal regions, has not been completely demonstrated.

Finally, improving performance in one task actually may not necessarily represent cognitive enhancement. More comprehensive outcome measures are needed to assess the clinical significance of rTMS or tDCS in AD and appropriately powered studies with sound blinding procedures are necessary [43]. It seems unlikely that stimulations over a single brain area will contribute to a significant improvement of the cognitive status of AD patients, particularly those with more advanced stages of disease. Multiple-target stimulation protocols are necessary in order to overcome the multiple cognitive deficits characterizing moderate or severe AD.
